# Oxidative aging of plant protein-stabilized emulsions and its impact on carotenoid bioaccessibility

**DOI:** 10.1016/j.fochx.2026.104513

**Published:** 2026-09-24

**Authors:** Uyory Choe

**Affiliations:** Department of Food and Nutrition, College of Biomedical and Health Science, Konkuk University, Chungju 27478, Republic of Korea

**Keywords:** Carotenoids, Plant proteins, Emulsions, Oxidative aging, Lipid digestion, Bioaccessibility

## Abstract

Plant proteins are widely used as natural emulsifiers for carotenoid-enriched foods, yet most delivery studies evaluate freshly prepared emulsions instead of products stored before consumption. During storage, lipids, interfacial proteins, and carotenoids undergo concurrent oxidation. Lipid hydroperoxides and secondary products can modify adsorbed proteins, while carotenoids are prone to isomerization, epoxidation, and oxidative cleavage. Accordingly, the interfacial layer encountered during digestion may differ markedly from that formed after homogenization. This difference can affect pepsin hydrolysis, bile salt displacement, lipase adsorption, lipid digestion, and carotenoid micellization. This review introduces oxidative aging as a concept for describing progressive chemical and structural changes in plant protein-stabilized oil-in-water emulsions during storage. It examines protein sources, residual non-protein components, degree-dependent protein oxidation, and their links to carotenoid bioaccessibility. Limitations of current storage and digestion studies are discussed, and a storage-to-digestion strategy is proposed to improve carotenoid protection during shelf life while maintaining gastrointestinal release.

## Highlights

• Oxidative aging alters plant protein interfaces and carotenoid bioaccessibility.
• Moderate protein oxidation may improve interfacial function and delivery.
• Severe oxidation forms rigid interfaces that restrict digestion and release.
• Residual metals and minor components strongly influence emulsion oxidation.
• Storage-to-digestion strategy is proposed for shelf-life-adjusted delivery.

## Graphical abstract

**Figure:**
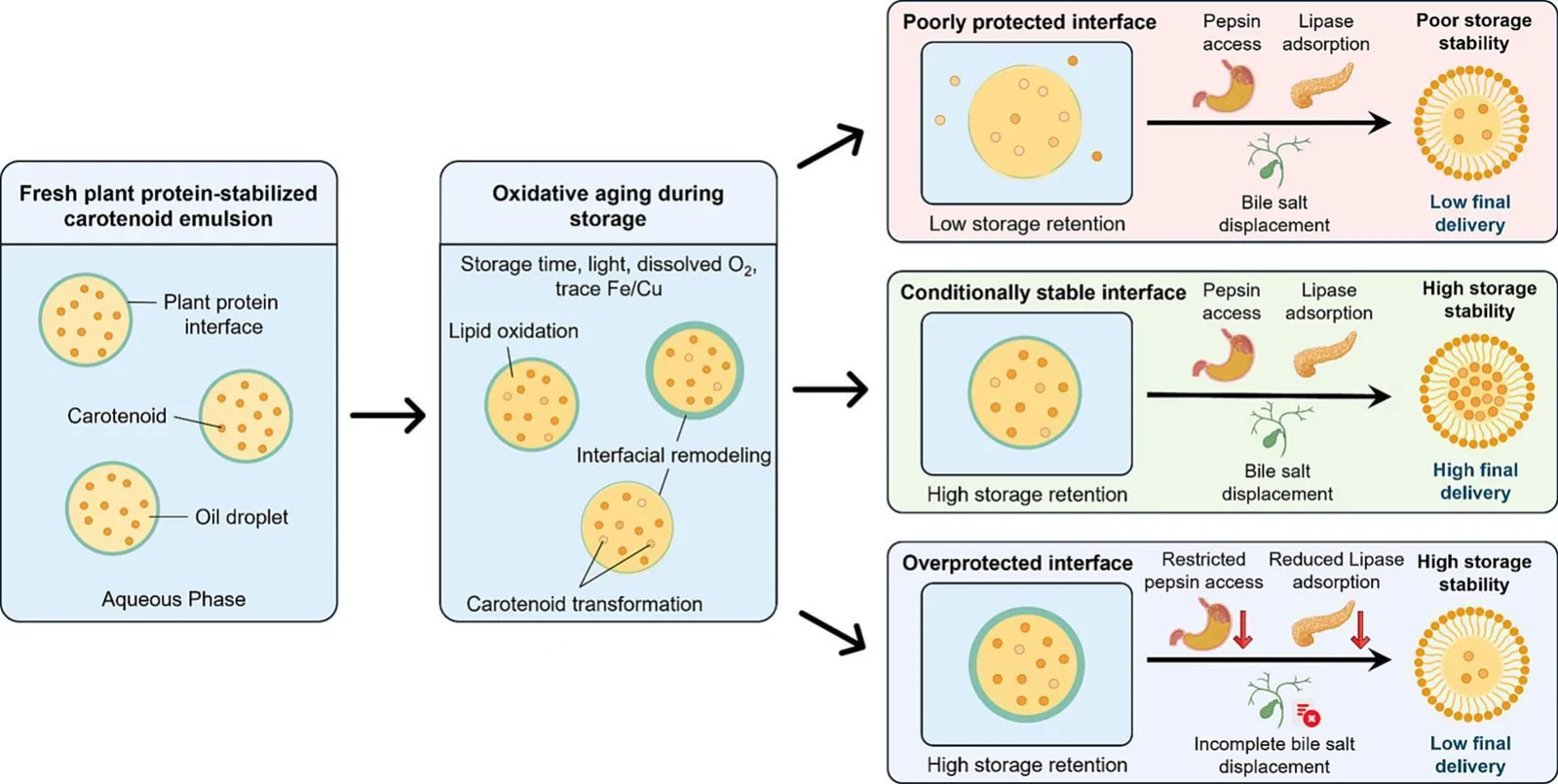

## 1 Introduction

Carotenoids are lipid-soluble pigments responsible for the yellow, orange, and red colors of many fruits, vegetables, algae, and microorganisms. β-Carotene, lycopene, lutein, zeaxanthin, and astaxanthin are commonly used in functional foods because of their coloring properties and biological activities (Sheng et al., 2026). However, their application in aqueous foods is limited by poor water solubility and low chemical stability. In particular, the conjugated double-bond system of carotenoids is easily affected by oxygen, light, heat, acidic conditions, and free radicals. These reactions can cause geometric isomerization, epoxidation, oxidative cleavage, and color loss during processing and storage (Meléndez-Martínez et al., 2023). Therefore, a high carotenoid concentration at the time of food production does not guarantee a high concentration at the time of consumption.

The nutritional use of carotenoids involves several steps. First, carotenoids must be released from the food or delivery matrix. The released carotenoids then need to remain in a digestible lipid phase. During intestinal digestion, triacylglycerols are converted into free fatty acids and monoacylglycerols. These digestion products interact with bile salts and phospholipids and form mixed colloidal structures. Carotenoids must be incorporated into these structures before they can approach the intestinal epithelium. Thus, bioaccessibility represents the fraction that becomes available for absorption after matrix release, lipid digestion, and micellization, rather than the fraction that simply remains after digestion (Geng, Bao, et al., 2023; Jalali-Jivan et al., 2022; Molteni et al., 2022; Xavier & Mercadante, 2022).

Oil-in-water emulsions have been used to improve carotenoid dispersibility and bioaccessibility. In these systems, carotenoids are dissolved or dispersed in the oil phase, and the oil is distributed as small droplets in water. The large interfacial area can support lipase adsorption and rapid lipid digestion. Also, droplet size, carrier oil, emulsifier type, electrical charge, and continuous-phase viscosity can be adjusted for a specific food application. Previous reviews have summarized the usefulness of emulsion-based delivery systems and interfacial engineering for carotenoid protection and release (Boonlao et al., 2022; Sereti et al., 2025; Yao et al., 2022; Zhao et al., 2026).

The interfacial area that promotes digestion can also increase chemical reactivity. Smaller droplets expose more lipid surface to water-soluble metals, oxygen, antioxidants, and proteins. If the adsorbed layer does not completely cover the droplets, lipid oxidation can be accelerated. On the contrary, a thick and strongly associated protein layer can reduce the access of prooxidants during storage. The same protein layer may also resist proteolysis and bile salt displacement during digestion. As one would expect, the interface that provides the best storage stability may not always provide the highest carotenoid bioaccessibility (Ma et al., 2022).

Plant proteins from legumes, cereals, oilseeds, and tubers have received increasing attention as alternatives to dairy and egg proteins. Soy, pea, faba bean, lentil, chickpea, rice, rice bran, oat, potato, and sunflower proteins contain both hydrophilic and hydrophobic regions. These proteins can adsorb at the oil-water interface, partially unfold, and form an interfacial protein layer around the oil droplets. Their use is especially suitable for dairy-free and plant-based foods. However, plant protein ingredients are not composed of protein alone. Depending on the raw material and fractionation method, protein isolates and concentrates may contain phenolic compounds, phytate, phospholipids, carbohydrates, endogenous lipids, pigments, and transition metals (Münch, Schroën, & Berton-Carabin, 2024).

These residual components can markedly change the oxidative stability of an emulsion. For example, phenolic compounds and phytate may show antioxidant effects, whereas soluble iron, copper, and pre-oxidized lipids may promote oxidation. Even when emulsions are prepared at the same protein concentration, commercial plant protein ingredients can show different oxidation rates because their soluble metal content and initial oxidation state are different (Münch, Stoyanov, et al., 2024). Therefore, the oxidative behavior of plant proteins cannot be predicted solely from protein purity or botanical source.

In contrast, dairy proteins such as whey protein and casein are generally used as more compositionally defined emulsifiers, and their oxidative behavior has been studied under relatively controlled conditions (Kim et al., 2020). Compared to dairy proteins, the oxidative response of plant protein ingredients is often more difficult to predict because it reflects not only protein structure but also processing history and residual non-protein components (Münch, Schroën, & Berton-Carabin, 2024).

Another important issue is that storage and digestion have generally been studied separately. In storage studies, lipid hydroperoxides, secondary oxidation products, droplet size, and carotenoid retention are often measured. In digestion studies, freshly prepared emulsions are usually used to determine free fatty acid release and carotenoid micellization. However, the digestive enzymes in an actual product do not encounter the fresh interface. Instead, they encounter an interface that may have been oxidized and reorganized during storage. Therefore, fresh-emulsion digestion data may not fully represent the behavior of a stored food product. In practice, this disconnect can lead to overestimation of carotenoid delivery at the time of consumption because a formulation showing high carotenoid bioaccessibility when freshly prepared may show lower bioaccessibility after storage.

Oxidation in protein-stabilized emulsions is not limited to the lipid phase. Lipid radicals, hydroperoxides, and reactive aldehydes can modify amino acid side chains and promote protein carbonylation, sulfhydryl loss, disulfide bond formation, fragmentation, and covalent aggregation. At the same time, carotenoids can initially quench reactive species but can become oxidation substrates as the radical concentration increases. As a result, lipids, proteins, and carotenoids form an interconnected reaction system at and near the oil-water interface (Hennebelle et al., 2024).

The effect of protein oxidation is also dependent on its degree. Studies of soy protein isolate and rice bran protein emulsions show that different oxidation levels produce different structural and functional responses (Chen et al., 2013; Li et al., 2021; Zhou et al., 2023). These studies suggest that oxidation should be considered as a continuous process rather than a simple comparison between oxidized and non-oxidized proteins.

In the current review, the term oxidative aging is used to describe the progressive chemical and physical modification of carotenoid-containing emulsions during storage. This concept includes lipid oxidation, protein oxidation, carotenoid transformation, changes in interfacial organization, and altered digestive disassembly. The review is focused mainly on oil-in-water emulsions and nanoemulsions stabilized by plant proteins. β-Carotene, lycopene, lutein, zeaxanthin, and astaxanthin are discussed where appropriate. The aim is to understand how plant protein interfaces are formed and how the interfaces change during storage. The review also examines how these changes influence protein digestion, lipid hydrolysis, and carotenoid micellization. Furthermore, storage-to-digestion is proposed as a new evaluation strategy in which emulsion samples are analyzed during storage and then subjected to gastrointestinal digestion at selected storage times. This strategy may provide a useful basis for designing future studies on the storage stability and digestive behavior of plant protein-stabilized carotenoid emulsions.

## 2 Oxidation-sensitive interfaces formed by plant proteins

### 2.1 Interfacial assembly of plant proteins

The formation of a protein-stabilized emulsion involves several consecutive events. Protein molecules or soluble protein aggregates first move from the aqueous phase to the newly formed oil-water interface. They then adsorb, lower the interfacial tension, and rearrange before the droplets recoalesce. During adsorption, hydrophobic amino acid regions tend to orient toward the oil phase, whereas hydrophilic and charged regions remain exposed to water. The final interfacial protein layer stabilizes droplets by electrostatic repulsion, steric effects, and interfacial viscoelasticity (Drusch et al., 2021).

Plant proteins show considerable differences in molecular structure. Legume proteins are commonly rich in 7S vicilin-type and 11S legumin-type globulins, while cereal proteins contain different proportions of albumins, globulins, prolamins, and glutelins. Potato protein contains patatin and protease-inhibitor fractions, whereas oilseed proteins may remain associated with phospholipids and phenolic compounds. These differences affect molecular size, flexibility, surface hydrophobicity, charge, and thermal stability. Consequently, plant proteins do not adsorb or reorganize at the oil-water interface in the same manner (Grasso et al., 2022; Liu, Yan, et al., 2024; Olsmats & Rennie, 2024; Wen et al., 2023).

Protein solubility is one of the major factors determining interfacial adsorption. Large insoluble aggregates move slowly and may not cover the new interface during homogenization. By contrast, small peptides can move rapidly but may form a thin interfacial layer with weak steric protection. Therefore, the effective emulsifying fraction of a plant protein ingredient is not equal to its total protein content. It includes only the proteins, peptides, and soluble aggregates that remain dispersed under the selected pH, ionic strength, and processing conditions (Drusch et al., 2021; Olsmats & Rennie, 2024).

After adsorption, the protein structure continues to change. Partial unfolding can expose hydrophobic residues and increase protein-oil interactions. In the meantime, hydrogen bonds, electrostatic interactions, hydrophobic interactions, and disulfide exchange can strengthen protein-protein associations within the interfacial protein layer. A moderate level of association can prevent droplet coalescence. However, extensive aggregation in the aqueous phase reduces the amount of protein available for adsorption, while excessive cross-linking at the interface may produce an interfacial protein layer that is difficult to digest (Costa et al., 2022; Zhang, Ning, et al., 2024).

The initial protein coverage is also important for chemical stability. A high interfacial protein concentration may provide more radical-scavenging amino acids and a thicker barrier between the aqueous phase and the oil. When the same protein amount is distributed over smaller droplets, the total surface area increases and the protein coverage per unit area may decrease. Therefore, the effect of droplet size on oxidation should be interpreted together with interfacial protein concentration and protein loading.

Proteins remaining in the continuous phase can also influence oxidation. They may bind transition metals and react with radicals before these species reach the droplets. On the contrary, soluble oxidized aggregates may carry metals or reactive lipid products between droplets and contribute to bridging flocculation. Thus, both adsorbed and non-adsorbed protein fractions need to be considered when explaining physical and oxidative stability.

Most carotenoid delivery studies report droplet size, ζ-potential, encapsulation efficiency, and creaming stability. These parameters are useful, but they do not directly describe the chemistry or mechanical strength of the interfacial protein layer. Measurements of adsorbed protein, interfacial tension, dilatational rheology, and oxidation of the interfacial protein are less common. It is worth noting that an emulsion can maintain a similar droplet size while its interfacial protein becomes extensively modified during storage (Hu et al., 2024).

### 2.2 Protein source-dependent susceptibility to oxidation

Protein oxidation can be caused by reactive oxygen species, metal-catalyzed reactions, and secondary lipid oxidation products. Cysteine and methionine are readily oxidized because of their sulfur-containing side chains. Tryptophan, tyrosine, phenylalanine, histidine, lysine, arginine, and proline can also undergo radical-mediated modification or carbonyl formation. The extent of oxidation depends not only on amino acid composition but also on whether these residues are exposed after unfolding and interfacial adsorption. Accordingly, differences in molecular structure and amino acid composition among plant protein sources can lead to differences in oxidation susceptibility by changing the abundance and accessibility of oxidation-sensitive residues. For example, in native globular proteins, some oxidation-sensitive residues can be buried within the folded structure and physically shielded from oxidants. Unfolding during processing or interfacial adsorption can expose these residues and increase their susceptibility to oxidation (Poojary & Lund, 2022).

Protein oxidation can alter the structural properties that determine the interfacial functionality of proteins, and the direction of these changes depends on the extent of oxidation. Limited cleavage of intramolecular interactions can increase flexibility and expose hydrophobic regions. This may improve diffusion and adsorption at the interface. In contrast, extensive oxidation can promote disulfide bonds, dityrosine formation, lipid aldehyde adducts, and high-molecular-weight aggregates. These products often have lower solubility and reduced molecular flexibility (Poojary & Lund, 2022).

The degree-dependent effect was clearly observed in soy protein isolate. Moderate peroxyl-radical oxidation changed the protein conformation and improved selected emulsifying properties, whereas higher oxidation levels promoted aggregation and decreased emulsion stability (Chen et al., 2013). A similar pattern was found in rice bran protein. Moderate rancidity-induced oxidation increased the flexibility of interface-adsorbed proteins and improved emulsion stability, but excessive oxidation reduced flexibility and weakened the emulsion (Li et al., 2021).

Further investigation of rice bran protein showed that oxidation changed adsorption kinetics, interfacial pressure, dilatational properties, and emulsion rheology. Moderate oxidation increased the diffusion rate and interfacial modulus, while severe oxidation enhanced aggregation and reduced favorable interfacial behavior (Li, Cai, et al., 2022). These results demonstrate that protein carbonyl content by itself is not sufficient to explain functionality. The structural location and extent of cross-linking are also important.

Botanical sources can influence oxidation susceptibility. Proteins rich in sulfur-containing and aromatic amino acids may provide radical-scavenging sites but can also be preferentially modified. Compact globulins may initially protect sensitive residues, but adsorption-induced unfolding exposes these residues near the lipid phase. Proteins that adsorb as aggregates can produce a thick interfacial protein layer, but the same aggregates may retain catalytic metals and create uneven regions at the droplet surface.

Processing and storage history further affect protein oxidation. Commercial ingredients may be exposed to alkaline extraction, acid precipitation, washing, membrane separation, heat treatment, spray drying, and storage prior to distribution. Harsh pH conditions can modify lysine and cysteine, and drying can form aggregates that only partly redisperse. During storage, exposure to oxygen, light, and heat can promote protein oxidation. Accordingly, differences attributed to soy, pea, rice, or faba bean proteins may arise not only from botanical origin but also from extraction severity and ingredient storage (Gultekin Subasi et al., 2022; Münch, Schroën, & Berton-Carabin, 2024).

Considering these effects, commercial plant protein ingredients may already undergo some protein denaturation and oxidation during processing and storage before they are used to prepare emulsions. This pre-existing oxidation is different from the oxidation that occurs after the proteins adsorb at the oil-water interface and the emulsion is stored. For this reason, studies using proteins that were oxidized before emulsification should be interpreted separately from studies examining oxidation that develops during storage of the final emulsion.

For a meaningful comparison, the oxidative and structural state of the protein should be determined before emulsion preparation. Protein carbonyls, free sulfhydryl groups, solubility, particle size, and surface hydrophobicity are useful minimum measurements. If possible, the adsorbed protein fraction should also be evaluated after homogenization because the proteins selected at the interface can differ from those remaining in the continuous phase.

### 2.3 Roles of residual non-protein components

Plant protein ingredients contain variable amounts of non-protein compounds. Some are naturally present in seeds, while others are concentrated or generated during processing. Although their concentrations can be low, they can strongly influence oxidation because many reactions are initiated by trace levels of transition metals and surface-active oxidation products.

Iron and copper are major prooxidants in emulsions because they decompose lipid hydroperoxides into reactive radicals. Their activity depends on solubility, oxidation state, ligand binding, and location. Fe^2+^ and Cu^+^ are the reduced forms that readily react with lipid hydroperoxides, whereas Fe^3+^ and Cu^2+^ can be converted back to these reduced forms through redox cycling. Free metal ions are generally more available for these reactions, while complexation with ligands can reduce or alter their catalytic activity depending on the ligand type and binding strength. For example, metals strongly bound within insoluble protein aggregates may show limited catalytic activity, whereas soluble metals located near the interface can rapidly promote oxidation. Therefore, total metal content does not fully reflect the prooxidant effect of iron and copper because their chemical form, location, and availability for redox reactions also determine their activity (Daoud et al., 2021; Hennebelle et al., 2024).

Phytate and phenolic compounds can have both protective and adverse effects. Phytate can chelate iron, although the effect depends on pH and the metal-to-phytate ratio. Phenolic compounds can scavenge radicals but may also reduce metal ions or form reactive quinones that interact with proteins. As a result, high levels of phytate or phenolic compounds do not always indicate high oxidative stability (Huang, Zeng, et al., 2022; Soendjaja & Girard, 2024).

Residual phospholipids and endogenous lipids can also modify the interfacial layer. Phospholipids may compete with proteins for adsorption and increase interfacial flexibility, while unsaturated phospholipids can also serve as oxidation substrates. Pre-oxidized endogenous lipids may introduce hydroperoxides and aldehydes into the emulsion before the carrier oil is added (Hennebelle et al., 2024; Shen et al., 2023).

Carbohydrates and fiber residues influence viscosity, protein solubility, and droplet interactions. Soluble polysaccharides can reduce droplet movement by increasing continuous-phase viscosity but may also promote depletion flocculation under some conditions, while insoluble particles may associate with the interface (He et al., 2020; McClements, 2000). Reducing sugars can also promote protein glycation during heating and alter protein properties (Poojary & Lund, 2022).

The practical importance of these components was demonstrated by comparing emulsions prepared with commercial soy and pea protein ingredients. Despite similar protein concentrations, differences in soluble iron and copper, phenolic content, and initial protein oxidation were associated with different lipid oxidation rates and oxidative stability (Münch, Schroën, & Berton-Carabin, 2024). This result indicates that a highly purified protein isolate is not necessarily a better antioxidant emulsifier than a less purified concentrate (Boerkamp et al., 2024).

The fractionation method should therefore be reported as a major characteristic of the ingredient. Dry fractionation retains more of the native seed matrix, whereas wet isolation can expose proteins to extreme pH, oxygen, and drying. Washing and defatting may remove prooxidants but can also remove natural antioxidants or leave oxidation products. These opposing changes explain why protein purity cannot fully predict emulsion oxidation (Münch, Stoyanov, et al., 2024).

For carotenoid delivery, residual components can affect storage and digestion at the same time. They may influence lipid oxidation, react with carotenoids, modify the interfacial protein, or interact with bile salts and digestive enzymes. Therefore, plant protein ingredients should be considered as multicomponent materials that contain both proteins and residual non-protein components. Future studies should report protein purity, residual lipid, soluble metals, phenolic compounds, phytate, phospholipids, and initial oxidation markers whenever possible.

## 3 Coupled oxidative aging of lipids, proteins, and carotenoids

Lipid oxidation, protein oxidation, and carotenoid degradation are often measured as separate events. However, this separation does not reflect the chemical environment of a protein-stabilized emulsion. Lipid radicals and aldehydes can oxidize proteins or form covalent adducts with amino acid residues; oxidized proteins can alter metal binding and radical reactions; and carotenoids can quench radicals or become oxidation substrates. Therefore, the oxidation of one component can alter the reactivity and location of the other components (Chen, Hu, et al., 2022; Hennebelle et al., 2024).

The oil-water interface is a major reaction region because it contains lipids, adsorbed proteins, surface-active minor compounds, and water-soluble prooxidants. Nevertheless, oxidation does not occur uniformly at the surface of every droplet. Reactive compounds can be produced in the oil core, at the interface, or in the continuous phase, and some of them can migrate between these regions. Spatial imaging has shown that protein and lipid oxidation can differ among droplets in concentrated legume protein emulsions (Brüls-Gill et al., 2024).

### 3.1 Initiation and propagation of lipid oxidation

Lipid oxidation starts when a hydrogen atom is removed from an unsaturated fatty acid and a lipid radical is formed. The radical reacts rapidly with oxygen and becomes a lipid peroxyl radical. This radical removes hydrogen from another lipid and produces a lipid hydroperoxide. Hydroperoxides can accumulate during the early stage of storage, but they are unstable in the presence of heat, light, iron, and copper. Their decomposition forms new radicals and secondary products such as aldehydes, ketones, alcohols, hydrocarbons, epoxides, and short-chain acids (Hennebelle et al., 2024).

In an emulsion, the radical-chain reaction is affected by the distribution of reactants. Metal ions and reducing compounds are often present in the aqueous phase, whereas unsaturated fatty acids are mainly located in the oil droplets. The interfacial layer controls how closely these reactants approach one another. Thus, oxidation depends on fatty acid unsaturation and oxygen concentration as well as interfacial composition, protein coverage, and the position of prooxidants and antioxidants (Daoud et al., 2021; Hennebelle et al., 2024).

The initial oxidative quality of the carrier oil is an important variable. Oil can contain hydroperoxides because of extraction, refining, transportation, heating, or previous storage. Homogenization distributes these products among many small droplets and exposes them to metals introduced with the protein ingredient, salts, or processing water. Therefore, two oils with a similar fatty acid composition can show different oxidation induction periods when their initial hydroperoxide levels are different (Hennebelle et al., 2024; Schroën & Berton-Carabin, 2022).

The major initiation pathway can also vary with emulsifier type. A kinetic study suggested that hydroperoxide decomposition was a major source of radicals in emulsions stabilized by low-molecular-weight surfactants. In protein-stabilized emulsions, reactions involving proteins contributed more strongly to radical initiation (Schroën & Berton-Carabin, 2022). This observation suggests that proteins are not only physical barriers or radical scavengers but can also participate directly in the oxidation network.

Transition metals are especially important in plant protein emulsions. Iron and copper may originate from the crop, processing equipment, water, or salts. Their activity is determined by soluble and weakly bound fractions instead of total content. For instance, differences in oxidation among soy, hydrolyzed soy, pea, and faba bean protein emulsions were associated with differences in the availability of transition metals introduced by the protein isolates (Brüls-Gill et al., 2024). Taken together, the oxidative behavior of plant protein emulsions is determined by both the properties of the interfacial protein layer and the availability of transition metals associated with the protein ingredient (Daoud et al., 2021).

In addition to the effects of interfacial proteins and transition metals, droplet size can influence oxidation because smaller droplets create a larger total oil–water interfacial area, increasing the area where interfacial lipids and lipid hydroperoxides can interact with aqueous-phase prooxidants such as transition metals. However, this relationship is not always linear. A larger interface can increase contact with prooxidants when the surface is incompletely covered. On the other hand, it can distribute metal-binding proteins and antioxidants over a greater area. In addition, droplet size changes with homogenization intensity and protein loading. Therefore, the oxidation effect of droplet size cannot be separated easily from interfacial protein packing and protein conformation (Aslam & Schroën, 2023).

Furthermore, oxidation reactions can also occur in the continuous phase and influence the overall oxidative behavior of the emulsion. Unadsorbed proteins may bind metals or react with lipid aldehydes before the lipid aldehydes reach the interface (Hennebelle et al., 2024; Poojary & Lund, 2022). During emulsification, tocopherols from the oil phase can migrate into these aggregates, where they influence oxidation together with transition metals (Brüls-Gill et al., 2024). These interactions can change the amounts of prooxidants and reactive lipid products reaching the interface and can indirectly influence the oxidation of interfacial proteins and carotenoids. These effects can be more important in concentrated emulsions because the distance between droplets is short. As a result, local oxidation conditions can be different even when the bulk composition is uniform.

Because lipid oxidation proceeds through multiple stages and can vary locally within an emulsion, it should not be evaluated using a single marker. Hydroperoxides may decrease after their decomposition rate becomes greater than their formation rate. Secondary oxidation products, including aldehydes, represent later stages of lipid oxidation, but some of these products can further react with proteins. Therefore, primary products, secondary products, initial oil quality, oxygen consumption, and protein oxidation should be considered together (Chen, Cao, et al., 2022).

### 3.2 Oxidative modification of interfacial proteins

Proteins can be oxidized directly by reactive oxygen species and indirectly by lipid oxidation products. Direct oxidation changes susceptible amino acid residues and can produce carbonyl groups, oxidized sulfur residues, dityrosine, peptide-chain cleavage, and altered secondary structure. Indirect oxidation occurs when electrophilic lipid aldehydes form covalent adducts with nucleophilic amino acid residues (Chen, Hu, et al., 2022; Feng et al., 2022).

Lysine, histidine, cysteine, arginine, and other nucleophilic residues can react with lipid-derived carbonyl compounds. In Schiff-base formation, the carbonyl group of an aldehyde reacts with a primary amino group, such as the ε-amino group of lysine or an N-terminal amino group, followed by the loss of water to form an imine (C=N) bond. Dialdehydes such as malondialdehyde can participate in Schiff-base reactions with amino groups and may promote cross-linking between amino acid residues or protein molecules. In Michael addition, nucleophilic groups, such as the thiol group of cysteine, the imidazole group of histidine, or the amino group of lysine, attack the β‑carbon of an α,β-unsaturated aldehyde and form a covalent adduct. Therefore, an increase in protein carbonyl content may originate from both direct amino acid oxidation and the addition of lipid-derived carbonyls (Domingues et al., 2013).

Interfacial proteins can be more susceptible to oxidation because they are located close to oxidizing lipids. Adsorption at the oil–water interface can also partially unfold proteins and expose oxidation-sensitive residues that were buried in the native structure, further increasing their susceptibility to oxidation. Close contact between adsorbed protein molecules can promote intermolecular cross-linking during oxidation.

Oxidation changes interfacial function through several mechanisms. Sulfhydryl loss and disulfide formation increase protein-protein association. Modification of charged residues changes electrostatic repulsion and hydration. Limited backbone cleavage may increase flexibility, while covalent cross-linking produces rigid aggregates. Surface hydrophobicity may increase as buried regions are exposed, but excessive aggregation can reduce solubility and adsorption (Gong et al., 2022).

The effect of protein oxidation on interfacial function is strongly dependent on the degree of oxidation. Moderate oxidation of soy protein produced soluble and flexible aggregates that improved selected emulsifying properties, whereas severe oxidation formed insoluble aggregates and decreased functionality (Chen et al., 2013). Importantly, moderate oxidation cannot be defined by a specific quantitative range because the oxidation method, protein source, and oxidation marker differ among studies. For example, Li et al. (2021) classified rice bran protein prepared after rice bran was stored for 1 day at 25 °C and 85% relative humidity as moderately oxidized, whereas protein prepared after 5–10 days of storage represented excessive oxidation. Protein carbonyl content increased from 1.76 nmol/mg in fresh rice bran protein to 18.67 nmol/mg after 10 days of rice bran storage. In contrast, Zhou et al. (2023) found the most favorable β-carotene delivery with rice bran protein obtained from rice bran stored for 5 days. Thus, moderate oxidation should be defined within each experimental system using quantitative oxidation markers, such as protein carbonyl content and free sulfhydryl content, together with structural and functional responses.

The degree-dependent effects of protein oxidation are also important for carotenoid delivery. Emulsions stabilized with moderately oxidized rice bran protein showed higher β-carotene retention, digestive stability, and cellular uptake than emulsions prepared with less or more extensively oxidized protein (Zhou et al., 2023). A related high-internal-phase Pickering emulsion study also found that moderate protein oxidation improved β-carotene stability, free fatty acid release, and bioaccessibility, whereas excessive oxidation reduced these effects (Zhao et al., 2024).

It is worth noting that these studies mainly used proteins oxidized before emulsion preparation. A pre-oxidized protein can unfold or aggregate before it reaches the interface. This differs from oxidation occurring after the protein has already adsorbed around a droplet. During storage of the final emulsion, lipid radicals and aldehydes are generated directly next to the constrained interfacial protein layer. Thus, pre-oxidation and storage-induced co-oxidation should not be assumed to have the same effect.

Because these two oxidation pathways modify proteins at different stages and locations, changes in conventional oxidation markers do not necessarily indicate how interfacial properties have changed. For example, carbonyl content may increase without a detectable change in droplet size, while a small change in sulfhydryl groups may substantially alter interfacial protein layer elasticity. Therefore, protein oxidation markers such as carbonyl content and sulfhydryl content should be evaluated together with molecular-weight distribution, protein adsorption, interfacial rheology, and microstructural observations. Representative quantitative studies examining plant protein oxidation and interfacial properties in relation to emulsion stability, digestion, and carotenoid delivery are summarized in Table 1.

**Table 1:** Representative quantitative studies linking plant protein oxidation and interfacial properties to carotenoid stability and digestive delivery.

Carotenoid	Protein/emulsion system	Treatment/design	Quantitative outcome	Stability/delivery outcome	Reference
–	Soy protein isolate O/W emulsion	AAPH-induced protein oxidation before emulsification	Carbonyls: 5.78 nmol/mg (control) → ↑ with oxidation	Moderate oxidation: ↑ flexibility/emulsifying properties; extensive oxidation: ↑ aggregation, ↓ stability	Chen et al. (2013)
–	Rice bran protein O/W emulsion	Rice bran storage: 0–10 d before protein extraction	Carbonyls: 1.76 → 18.67 nmol/mg; protein size: 24.36 → 18.17 nm (1 d), 37.84–68.06 nm (5–10 d)	Lower oxidation: ↑ interfacial properties/stability; extensive oxidation: ↑ aggregation, ↓ stability	Li et al. (2021); Li et al. (2022)
β-Carotene	Rice bran protein O/W emulsion	Different protein oxidation levels before emulsification	Droplet size: 510 nm → 2149 nm (gastric) → 998 nm (intestinal); gastric retention: +23.50%; cellular uptake: +13.84%	Intermediate oxidation: ↑ β-carotene retention and cellular uptake	Zhou et al. (2023)
β-Carotene	Rice bran protein fibril high-internal-phase Pickering emulsion	Rice bran storage: 0–10 d; fibril formation: 90 °C, 2–12 h	Physical stability: up to 56 d at 25 °C	Intermediate oxidation: ↑ FFA release, ↑ bioaccessibility; extensive oxidation: ↓ both	Zhao et al. (2024)
β-Carotene	Wheat-, pea-, and soy-protein O/W emulsions	Protein-source comparison; pH 3.0 and 7.0	Bioaccessibility: ∼9–13%; wheat: 12.61 ± 1.03% (pH 3), 13.41 ± 4.09% (pH 7)	Wheat > pea/soy for β-carotene bioaccessibility	Ramezani et al. (2026)
Lutein	Walnut protein isolate O/W emulsion	Untreated *vs.* ultrasound-, heat-, and enzyme-modified protein	Encapsulation: 49.03 ± 1.47% → 79.96 ± 1.29%; maximum digestive retention: 49.67 ± 1.10%; maximum bioaccessibility: 40.49 ± 1.29%	Protein modification: ↑ lutein retention and bioaccessibility; oxidation not independently controlled	Liu et al. (2024)
Lycopene	Alginate/soy protein isolate emulsion gel beads	Formulation pH: 3.0, 5.0, and 7.0	Bioaccessibility: ∼0–12%	Highest bioaccessibility at pH 3.0; oxidation not independently controlled	Lin et al. (2022)
Lycopene	Walnut protein isolate–EGCG nanoparticles	Ultrasound-assisted protein–polyphenol carrier formation	Loading efficiency: 20.21%; particle size: 121.72 → 208.14–230.22 nm after lycopene loading	↑ Lycopene stability; gastrointestinal bioaccessibility not evaluated	Wang et al. (2024)

AAPH, 2,2′-azobis(2-amidinopropane) dihydrochloride; EGCG, epigallocatechin gallate; FFA, free fatty acids; O/W, oil-in-water; ↑, increase; ↓, decrease.

### 3.3 Carotenoid degradation and structural transformation

The conjugated double-bond system of carotenoids is responsible for both their color and chemical reactivity. During storage, carotenoids can undergo *E*-Z isomerization, epoxidation, oxidative cleavage, and further fragmentation. The dominant pathway depends on molecular structure, oxygen, temperature, light, pH, carrier oil, and the radical environment (Li, Hu, et al., 2022; Meléndez-Martínez et al., 2023).

Carotenoids can react with singlet oxygen and lipid peroxyl radicals. At relatively low radical concentrations, carotenoids may act as antioxidants by scavenging these reactive species and slowing the propagation of lipid oxidation. However, the carotenoid radical can react with oxygen when the oxidative stress is high or when co-antioxidants are insufficient. A high oxygen partial pressure favors reactions between oxygen and carotenoid radicals, leading to the formation of carotenoid peroxyl radicals that can promote further oxidation. In addition, depletion of co-antioxidants such as tocopherol reduces the capacity of the system to remove lipid peroxyl radicals, increasing the radical burden on carotenoids. Therefore, carotenoids should not be described as antioxidants under all emulsion conditions. Their protective or degradative behavior depends on oxygen partial pressure, radical flux, carotenoid concentration, co-antioxidant availability, and location (El-Agamey et al., 2004; Young & Lowe, 2001).

In protein-stabilized emulsions, carotenoid degradation can also occur together with oxidation of the interfacial protein layer. In whey protein isolate (WPI) emulsions, conditions that promoted protein oxidation were associated with faster β-carotene loss. EDTA and α-tocopherol increased β-carotene stability, and radical scavenging was more effective than metal chelation under the tested conditions (Xu et al., 2013). These results indicate that β-carotene degradation in emulsions is linked to the surrounding oxidative reactions involving the interfacial protein and lipid phase.

However, the use of whey protein limits direct comparison with plant protein-stabilized emulsions. Plant proteins differ in molecular structure, amino acid composition, processing history, and residual non-protein components, all of which can influence interfacial oxidation. Thus, the WPI study is useful for understanding coupled protein–carotenoid oxidation, but further studies using plant protein-stabilized emulsions are needed to determine how these reactions occur in plant-based systems.

Beyond the type of interfacial protein, the location of each carotenoid within the emulsion can also influence its exposure to oxidative reactions. β-Carotene and lycopene are nonpolar and tend to remain in the oil interior. Lutein and zeaxanthin contain hydroxyl groups and can be located closer to the interface. Astaxanthin also contains polar oxygenated groups and may therefore be located closer to the interfacial region. Thus, xanthophylls may be more exposed to aqueous prooxidants but may also be located where radical interception is most effective (Molteni et al., 2022).

In addition to differences in their location within emulsions, carotenoids can undergo geometric isomerization, which should be distinguished from complete carotenoid destruction. *Z*-isomers can have different color, provitamin A activity, crystallinity, and micellar incorporation compared with all-E forms. A decrease in the amount of the all-E isomer does not always mean that the carotenoid has been completely lost. Thus, a total-carotenoid assay may not fully reflect changes in isomer composition (Meléndez-Martínez et al., 2023; Yang et al., 2018).

Unlike geometric isomerization, oxidative cleavage breaks the carotenoid structure and produces apocarotenoids with shorter carbon chains. Some products have aroma activity, while others indicate severe oxidation. Their increased polarity may allow migration from the oil core to the interface or water phase, where additional reactions can occur. In addition, these products may not be recovered by an extraction method designed for the parent carotenoid (Meléndez-Martínez et al., 2023).

For these reasons, absorbance loss or total carotenoid concentration is not sufficient to characterize these changes. HPLC should be used to resolve the parent carotenoid and major geometric isomers. When possible, LC-MS should be included to identify epoxides and apocarotenoids. A carotenoid mass balance is important when storage oxidation is linked to digestion because the compound entering the digestive system may already differ from that originally added.

### 3.4 Oxidative remodeling of the oil-water interface

The combined oxidation of lipids, proteins, and carotenoids progressively changes the identity of the oil-water interface. In the current review, this process is referred to as oxidative interfacial remodeling. It includes protein unfolding and cross-linking, accumulation of surface-active lipid oxidation products, carotenoid redistribution, changes in interfacial charge, and association of oxidized materials with neighboring droplets. The coupled progression from an initially dispersed emulsion to an oxidatively aged interfacial state is summarized in Fig. 1.

**Fig. 1:**
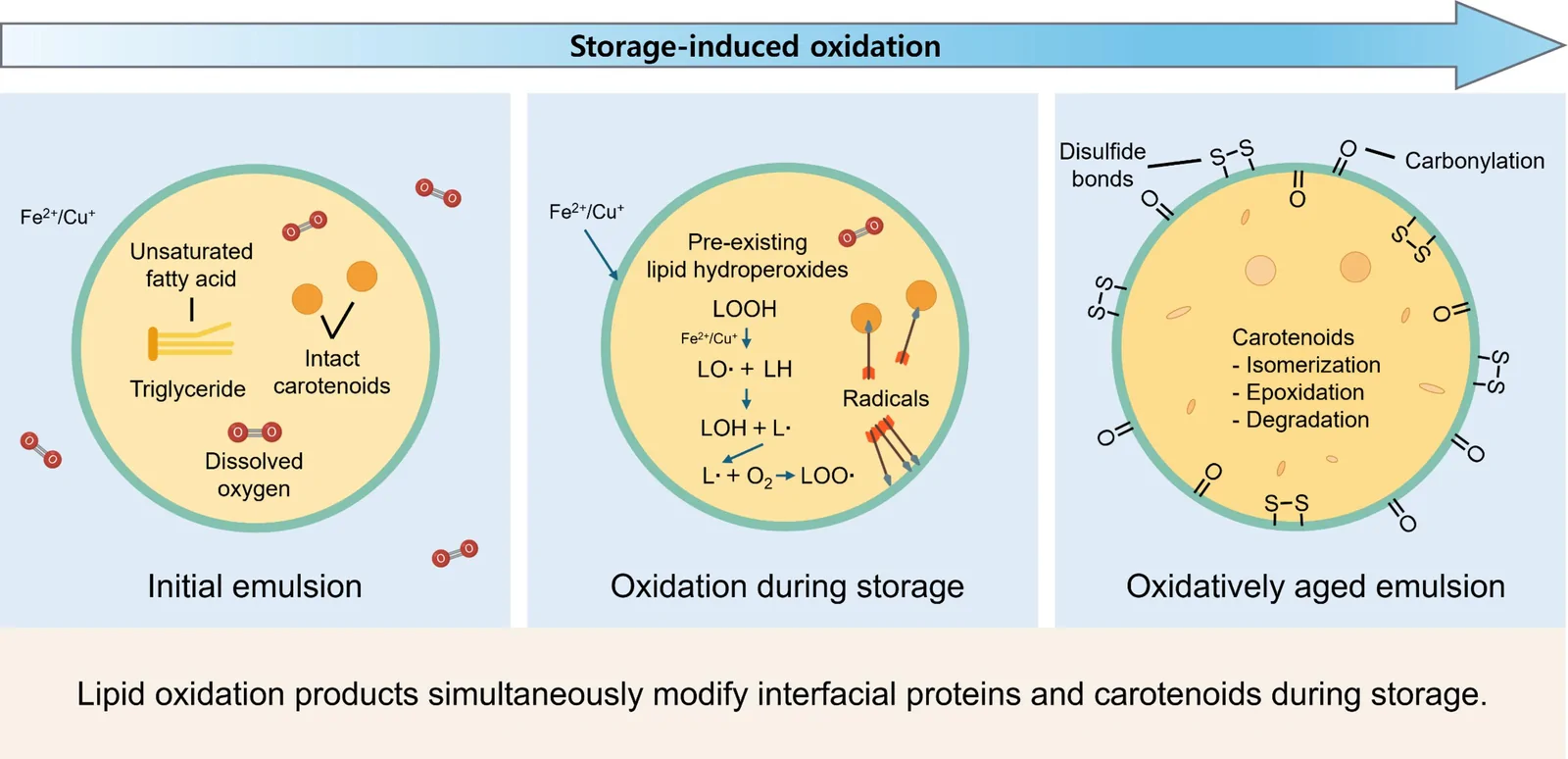
Storage-induced oxidative aging of carotenoid-loaded plant protein-stabilized emulsions. The initial emulsion contains unsaturated triacylglycerols, intact carotenoids, dissolved oxygen, aqueous Fe^2+^ and Cu^+^ ions, and a plant protein-stabilized oil–water interface. During storage, Fe^2+^ and Cu^+^ ions in the aqueous phase move toward the oil–water interface, where they catalyze the decomposition of pre-existing lipid hydroperoxides and initiate radical-chain reactions in the lipid phase. Continued oxidation modifies the interfacial protein through carbonylation and disulfide-bond formation, while carotenoids undergo isomerization, epoxidation, and oxidative degradation. These coupled reactions progressively transform the fresh interface into an oxidatively aged structure with altered chemical and digestive properties. LH, unsaturated lipid; L•, lipid radical; LO•, lipid alkoxyl radical; LOO•, lipid peroxyl radical; LOOH, lipid hydroperoxide.

During the early stage of storage, limited protein modification may strengthen a weak interface. Reactive amino acids can consume radicals, and unadsorbed proteins or phenolics can delay oxidation. Consequently, the droplet size and carotenoid concentration may remain relatively stable even though the chemistry of the interfacial layer has started to change (Chen et al., 2013; Hennebelle et al., 2024).

As oxidation continues, lipid hydroperoxides and aldehydes react with the adsorbed protein. Carbonylation and sulfhydryl oxidation alter hydration and charge, while protein-protein and protein-lipid cross-links increase rigidity. Oxidized phospholipids and amphiphilic secondary products may be incorporated into the interfacial layer. In the meantime, carotenoids can be depleted or converted into more polar compounds (Chen, Hu, et al., 2022; Hennebelle et al., 2024; Meléndez-Martínez et al., 2023).

At an advanced stage, cross-linked proteins may bridge droplets and form a continuous network. This can increase the apparent particle size and reduce the interfacial area available to digestive enzymes. Conversely, protein fragmentation and desorption may weaken the interfacial protein layer and promote coalescence. Which of these two mechanisms dominates depends on protein source, oxidation degree, pH, ionic strength, and the concentration of unadsorbed material (Chen et al., 2013; Hinderink et al., 2020).

Because physical changes do not always reflect the extent of chemical oxidation, visual appearance and droplet size should not be used as substitutes for chemical analysis. An emulsion can remain visually homogeneous and show a similar mean droplet size while protein carbonyls increase and carotenoids are degraded. In contrast, reversible flocculation can occur without extensive oxidation. Therefore, oxidative aging is not the same as creaming, flocculation, or coalescence, although these processes can interact (Hennebelle et al., 2024; Xu et al., 2013).

However, chemical measurements of the whole emulsion may not detect local differences in oxidation. In concentrated emulsions, some droplets can be oxidized more rapidly because of local metal concentration, antioxidant distribution, or oxygen exposure (Brüls-Gill et al., 2024). This indicates that some droplets can become highly oxidized even when the average oxidation level of the whole emulsion remains moderate, and these droplets can respond differently during digestion.

These differences in oxidation make the oxidative condition at the time of consumption more relevant to digestion than the initial condition after homogenization. This is because gastrointestinal conditions and digestive enzymes encounter the interfacial layer after it has been modified during storage. Therefore, the oxidative history of an emulsion should be included when interpreting protein digestion, lipid hydrolysis, and carotenoid micellization.

Overall, oxidative aging may follow a non-linear sequence. Mild changes can improve interfacial cohesion, intermediate changes can alter enzyme accessibility without visible destabilization, and severe changes can produce rigid aggregates or interfacial failure. This concept can explain why moderate protein oxidation sometimes improves carotenoid delivery while extensive oxidation reduces it. The coupled oxidative changes occurring in the lipid phase, interfacial proteins, carotenoids, minor ingredients, and droplet structure, together with their analytical and digestive implications, are summarized in Table 2 (Chen et al., 2013; Li et al., 2021; Li, Hu, et al., 2022; Zhou et al., 2023).

**Table 2:** Coupled oxidative changes in plant-protein-stabilized carotenoid emulsions and their analytical and digestive implications.

Emulsion component/region	Major oxidative changes	Interfacial/colloidal effects	Effects during digestion	Key measurements	References
Lipid phase	Lipid radicals/LOOH → aldehydes, ketones, epoxides, short-chain acids	↑ Surface-active oxidation products; altered interfacial packing; protein–carbonyl reactions	Altered lipase susceptibility; pH-stat interference; continued aldehyde–protein reactions	LOOH; secondary oxidation products; oxygen consumption; FFA/MAG/TAG	Hennebelle et al. (2024); Brüls-Gill et al. (2024)
Interfacial protein	Carbonylation; sulfhydryl loss; Met/Cys oxidation; disulfide/dityrosine formation; aggregation	Moderate oxidation: ↑ flexibility/cohesion; severe oxidation: rigid/insoluble protein layers	Moderate unfolding: ↑ pepsin accessibility; severe cross-linking: ↓ pepsin diffusion, bile displacement, lipase adsorption	Carbonyls; free sulfhydryls; SDS-PAGE/SEC; hydrophobicity; adsorbed protein; interfacial rheology	Chen et al. (2013); Li et al. (2021); Li et al. (2022); Zhao et al. (2023)
Carotenoids	E–Z isomerization; epoxidation; oxidative cleavage; apocarotenoid formation	Parent carotenoid loss/redistribution; ↑ interface-proximal polar products	Altered release, micellization, recovery, and uptake	HPLC isomers; LC-MS oxidation products; carotenoid mass balance	Mutsokoti et al. (2017); Meléndez-Martínez et al. (2023)
Minor ingredients and metals	Fe/Cu-catalyzed LOOH decomposition; phenolic redox reactions; phospholipid/endogenous lipid oxidation	Metal localization near interface; competition for adsorption; altered interfacial charge/flexibility	Altered proteolysis, bile interactions, colloid formation, and oxidation	Soluble Fe/Cu; phenolics; phytate; phospholipids; residual/pre-oxidized lipids	Daoud et al. (2021); Münch et al. (2024a); Münch et al. (2024b); Soendjaja and Girard (2024)
Interfacial and droplet structure	Changes in charge, hydration, protein packing, adsorption/desorption, bridging, and fragmentation	Flocculation; rigid networks; coalescence; interfacial failure	Altered enzyme accessibility and lipolysis	Droplet size; microscopy; ζ-potential; adsorbed protein; interfacial rheology; fraction-specific oxidation	Li et al. (2021); Li et al. (2022); Brüls-Gill et al. (2024)

FFA, free fatty acids; LOOH, lipid hydroperoxide; MAG, monoacylglycerols; SDS-PAGE, sodium dodecyl sulfate-polyacrylamide gel electrophoresis; SEC, size-exclusion chromatography; TAG, triacylglycerols. ↑, increase; ↓, decrease.

## 4 Consequences of oxidative aging for digestion and carotenoid bioaccessibility

After ingestion, the emulsion is exposed to dilution, changing pH, salts, digestive enzymes, bile components, and mechanical mixing. Before gastric digestion, the emulsion first comes into contact with saliva. Salivary components, including mucins and salts, can interact with protein-coated droplets and induce flocculation, which may change the aggregation state of the emulsion entering the stomach (Liu et al., 2025; Vingerhoeds et al., 2005). These conditions progressively remove or reorganize the interfacial protein layer and determine whether the lipid droplets can be hydrolyzed. However, a stored emulsion enters digestion with oxidized lipids, modified proteins, carotenoid isomers, and degradation products. Therefore, the digestive behavior of an aged emulsion can differ from that of a fresh emulsion.

The influence of oxidation on emulsion digestion is not expected to be linear. Limited oxidation can expose cleavage sites and weaken non-covalent interactions, while severe oxidation can form cross-linked layers that restrict enzyme transport. Extensive fragmentation can also destabilize the interface and expose the oil rapidly. The final response depends on the protein, oxidation pathway, carrier oil, and distribution of oxidized products. A time-resolved experimental workflow linking ingredient characterization, oxidative aging, gastrointestinal digestion, and fraction-specific carotenoid recovery is outlined in Fig. 2.

**Fig. 2:**
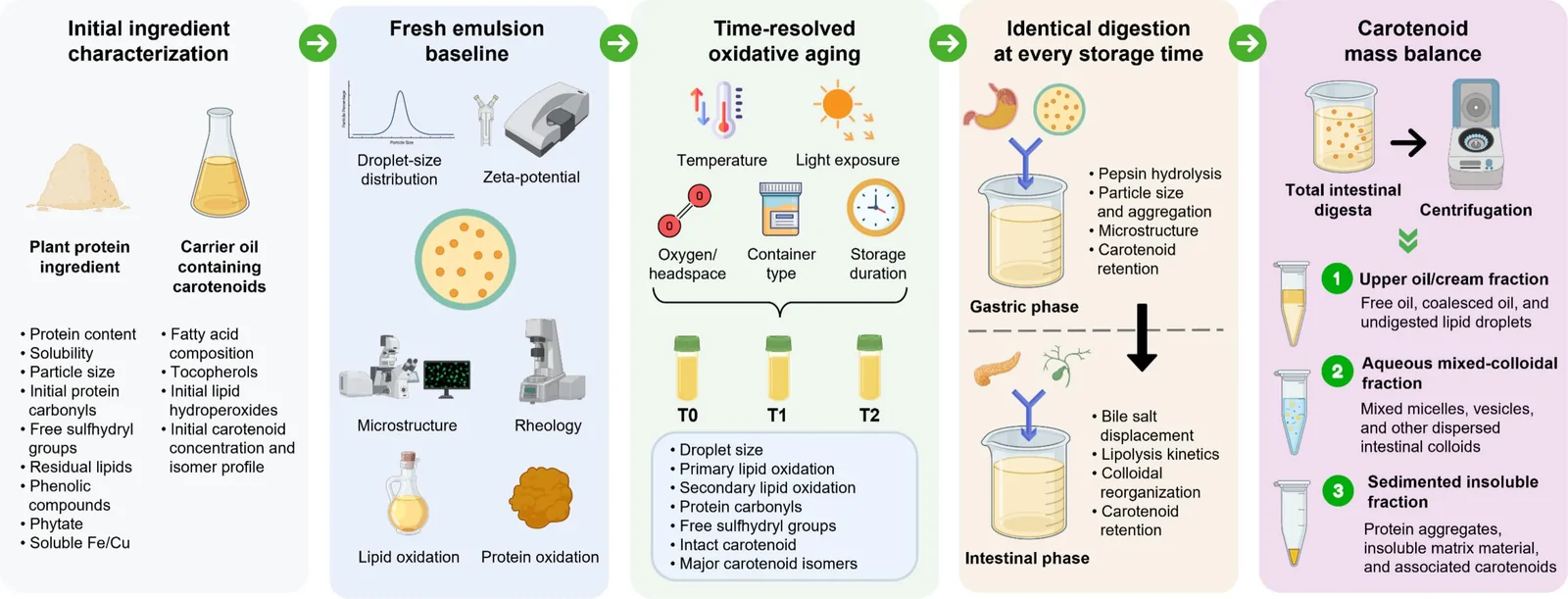
Time-resolved storage-to-digestion evaluation strategy for carotenoid-loaded plant protein-stabilized emulsions. The proposed workflow begins with characterization of the plant protein ingredient and carotenoid-containing carrier oil, followed by establishment of the fresh-emulsion baseline. Identical emulsion batches are then analyzed at multiple storage times under controlled temperature, light, oxygen, container, and storage-duration conditions. Samples collected at each time point undergo the same gastric and intestinal digestion procedures to determine protein hydrolysis, structural disassembly, lipolysis, and carotenoid retention. Following intestinal digestion, carotenoids are quantified in the total digesta and in the upper oil or cream fraction, aqueous mixed-colloidal fraction, and sedimented insoluble fraction to establish a fraction-specific mass balance.

### 4.1 Gastric destabilization and protein digestion

The gastric phase is the first major stage of emulsion disruption. The droplets are exposed to acidification, increased ionic strength, pepsin, mechanical movement, and gastric lipase. Many plant storage proteins have low solubility near the gastric pH range. As the pH approaches the isoelectric point, electrostatic repulsion is reduced and the protein-coated droplets can form flocs or larger aggregates (Iddir et al., 2021).

However, this acid-induced aggregation does not necessarily mean that droplet coalescence has occurred. Individual oil droplets may remain intact within a loose floc, or the interfacial protein layer may collapse and allow partial oil separation. These structures show different accessibility to pepsin. They also provide different surface areas for the following intestinal digestion stage.

Once pepsin reaches the interfacial protein layer, hydrolysis reduces its molecular size and strength. The newly formed peptides may desorb into the aqueous phase, but some can remain surface active and continue to stabilize the oil droplets. Therefore, disappearance of the original protein band does not necessarily mean that the interface is completely removed (Gao et al., 2024).

The extent of this pepsin hydrolysis can be altered by protein oxidation. Protein oxidation can mask cleavage sites, promote disulfide bond formation, and generate covalent aldehyde adducts. Dense aggregates can also restrict enzyme diffusion. On the other hand, moderate unfolding may expose peptide bonds and increase hydrolysis. Thus, the degree of hydrolysis should be interpreted together with particle size, microstructure, and soluble peptide release (Cheng et al., 2024).

In a recent study, Zhao et al. (2023) reported that increasing oxidation in soy protein isolate emulsions altered protein adsorption, droplet organization, and pepsin diffusion. Highly oxidized structures restricted pepsin transport and reduced gastric disintegration and protein hydrolysis, indicating that lower digestibility can result from both reduced molecular susceptibility to hydrolysis and limited enzyme access through the emulsion structure.

Plant protein sources also influence gastric behavior. Wheat, pea, and soy protein emulsions showed different acid stability and proteolysis during simulated digestion. Under the tested acidic conditions, wheat protein emulsions showed more favorable digestion and higher β-carotene bioaccessibility than pea and soy protein systems (Ramezani et al., 2026). Although fresh emulsions were used, the study demonstrates that the effect of oxidative aging must be interpreted in relation to the original gastric behavior of each protein (Rivera Del Rio et al., 2022).

These differences in gastric behavior should also be considered when interpreting carotenoid retention during gastric digestion. A high retention value can reflect either chemical protection or carotenoid entrapment in a dense, poorly digestible aggregate. Partial disruption of the emulsion structure during gastric digestion can release carotenoids from dense aggregates, reducing their apparent gastric retention while improving their accessibility during subsequent intestinal digestion. For this reason, gastric carotenoid retention should be interpreted together with lipid digestion and micellization during the intestinal phase.

To distinguish these possibilities, a rigorous evaluation should quantify the carotenoid before storage, immediately before digestion, and after gastric digestion. When large aggregates are formed, the gastric digesta should be divided into soluble, dispersed, and sedimented fractions. This approach can distinguish chemical degradation from physical entrapment.

### 4.2 Interfacial displacement and intestinal lipolysis

When gastric digesta enters the small intestine, it is neutralized and mixed with bile and pancreatic secretions. Bile salts and phospholipids adsorb to the droplet surface and displace or reorganize the original emulsifier. Pancreatic lipase, assisted by colipase, then gains access to triacylglycerols. The ease of this process depends on the remaining interfacial protein layer and the peptides formed during gastric digestion (Malaki Nik et al., 2011).

A loosely associated or extensively hydrolyzed interfacial protein layer can be displaced readily. In contrast, a covalently cross-linked interfacial protein layer may remain around the droplet and prevent lipase adsorption. Peptides and oxidized protein aggregates in the aqueous phase may also compete with bile salts or attach to the droplets. As a result, the chemical accessibility of the surface can be more important than the geometric surface area (Ji et al., 2022; Yan et al., 2022).

Because oxidation can modify this interfacial accessibility, its effect on lipolysis depends on the degree of oxidation. Moderate oxidation can increase lipase access if it promotes partial unfolding or weakens the interfacial protein layer. Excessive oxidation can decrease lipolysis by causing protein cross-linking and persistent flocculation. Large loose flocs can redisperse after neutralization, whereas dense aggregates may remain throughout the intestinal phase. Gastric coalescence can also reduce the total droplet area and slow lipolysis (Zhao et al., 2024).

Beyond the degree of oxidation, comparisons of fresh protein-stabilized emulsions have shown that the protein source can also influence digestive aggregation and free fatty acid release. β-Carotene nanoemulsions stabilized by peanut, soy, rice bran, and whey proteins exhibited different digestion behaviors and carotenoid release (Liu et al., 2021). However, it remains unclear whether the same ranking would be observed after storage-induced oxidation (Fitri et al., 2022; Fu et al., 2019; Wei et al., 2021; Xiao et al., 2024).

In addition to protein source, droplet size and interfacial structure can also influence lipolysis. A small initial droplet size does not always result in rapid lipolysis. A dense interfacial layer can restrict enzyme adsorption even when the surface area is large. Similarly, oxidation-induced flocculation can increase the measured particle size without reducing the actual surface area if individual droplets remain intact. Therefore, particle size data should be supported by microscopy and lipolysis kinetics (Lu et al., 2017; Salvia-Trujillo et al., 2013; Tan et al., 2021).

At the same time, lipid oxidation products may affect digestion directly. Oxidized triacylglycerols and phospholipids can have different surface activity and enzyme susceptibility. Reactive aldehydes can continue to modify proteins during digestion, and acidic oxidation products can influence pH-stat measurements. These possibilities are rarely considered in carotenoid delivery studies (Han et al., 2025).

Because these oxidation-related effects can alter both the rate and extent of lipid digestion, lipolysis should be measured as a kinetic process. Oxidized interfacial protein layers may increase the lag time, reduce the maximum rate, or lower the final extent of digestion. A single final free fatty acid value cannot distinguish these effects. It is therefore useful to report the lag phase, initial rate, and digestion plateau (Li et al., 2011).

The pH-stat method is widely used for this purpose, but it estimates lipid hydrolysis from alkali consumption. The measured free fatty acid release depends on the ionization state of fatty acids and can be affected by factors such as calcium concentration (Li et al., 2011). When oxidative aging is the main subject, pH-stat data should be supported by direct analysis of free fatty acids, monoacylglycerols, and residual triacylglycerols.

### 4.3 Release and micellization of carotenoids

Carotenoid bioaccessibility is commonly calculated from the amount recovered in the aqueous mixed-colloidal fraction after intestinal digestion. However, this value reflects several different events. The carotenoid must remain chemically intact, be released from the emulsion, avoid precipitation or sedimentation, and become incorporated into mixed micelles or related intestinal colloids.

These events should be evaluated separately. A carotenoid can remain in the total digesta but stay trapped in undigested oil droplets. It can also be released from the original droplet and then associated with insoluble protein. Therefore, digestive retention, matrix release, and micellization are not interchangeable terms.

Micellization is closely linked to lipid digestion because free fatty acids and monoacylglycerols contribute to mixed micelle formation. Nevertheless, the relationship is not always proportional. In soy protein emulsions, lipolysis facilitated transfer of lipophilic compounds, but β-carotene showed slower and lower transfer than vitamin D3 and phytosterols because of its greater hydrophobicity (Fernandes et al., 2021; Gomes et al., 2021; Lara-Abia et al., 2023; Nik et al., 2011; Zhang & Li, 2022).

Consistent with this, kinetic studies showed that fatty acid release can occur faster than mixed micelle formation. The incorporation rates of lycopene, α-carotene, and β-carotene were related to lipid digestion products, but the relationships were compound-dependent (Mutsokoti et al., 2017). Therefore, an emulsion with rapid lipid hydrolysis does not necessarily have a high carotenoid micellization efficiency (Salvia-Trujillo et al., 2017).

The food matrix can further influence the relationship between lipolysis and carotenoid bioaccessibility. When a β-carotene emulsion was incorporated into milk or yogurt, lipid hydrolysis increased relative to digestion of the same emulsion without incorporation into milk or yogurt, but β-carotene bioaccessibility did not increase proportionally (Molet-Rodríguez et al., 2025). This result demonstrates that matrix components may improve lipolysis while limiting micelle formation or carotenoid incorporation (Yang et al., 2023).

Oxidation of lipid digestion products can also complicate the relationship between lipolysis and carotenoid micellization. The introduction of hydroperoxide, hydroxyl, epoxy, or carbonyl groups increases the polarity of oxidized fatty acids and monoacylglycerols and may alter their surface activity, aggregation behavior, and micellar association (Abousalham et al., 2000; Verhagen et al., 1978). These changes may affect the interactions of oxidized fatty acids and monoacylglycerols with bile salts and phospholipids during mixed micelle formation and consequently influence carotenoid incorporation. However, direct quantitative information on these effects under gastrointestinal conditions remains limited.

More broadly, oxidative aging can lower the final micellar carotenoid content at several stages of the delivery process. Storage can reduce the amount of intact carotenoid (Meléndez-Martínez et al., 2023). Oxidized droplets can resist lipolysis (Zhao et al., 2024). The remaining carotenoid can associate with oxidized protein or partition poorly into micelles. Isomers and oxidation products can also show different analytical recovery. Therefore, the stage at which the delivery system fails should be identified.

Accurate identification of this stage also depends on how the mixed-colloidal fraction is isolated. Centrifugation is commonly followed by filtration, but filters can retain carotenoid-containing colloids and underestimate bioaccessibility (Liu et al., 2022). This effect was especially important for acylated lutein in a casein-stabilized emulsion (Mantovani et al., 2025). Oxidized protein aggregates may increase colloid size and make the loss greater in stored samples.

To improve the reliability of this analysis, the centrifugation force, time, temperature, filter material, pore size, and carotenoid recovery should be reported (Liu et al., 2022). Dynamic light scattering provides particle-size information but does not identify whether the detected colloids are mixed micelles, vesicles, protein aggregates, or small residual oil droplets. Hence, the use of size-exclusion chromatography, asymmetric-flow field-flow fractionation, cryo-TEM, or small-angle scattering can provide stronger evidence regarding the colloidal form of the carotenoid.

### 4.4 Carotenoid-specific responses

The influence of oxidative aging cannot be generalized to all carotenoids. β-Carotene is highly nonpolar and is usually located in the oil interior. Its micellization depends strongly on lipid hydrolysis and the capacity of the mixed micelles. It may be partly protected from aqueous prooxidants but remains susceptible to radicals generated from unsaturated lipids (Molteni et al., 2022; Qian et al., 2012).

Lycopene is also nonpolar, but its acyclic and highly conjugated structure makes it sensitive to isomerization and oxidation. Because of its high hydrophobicity, its transfer into mixed micelles is generally lower than that of more polar carotenoids. Accordingly, a reduction in lipid digestion or micellar capacity may affect lycopene more strongly than more polar carotenoids (Lin et al., 2022; Mutsokoti et al., 2017; Wang, Wu, et al., 2024; Zhu et al., 2024).

Carotenoid geometry can also influence micellization. *Z*-isomers can show greater apparent micellization than all-E forms because their bent structure reduces crystal packing. Faster micellar incorporation of Z-isomers has been observed during *in vitro* digestion (Mutsokoti et al., 2017). Therefore, storage-induced isomerization may reduce the original all-E concentration but does not always reduce micellization to the same extent.

Micellization is also influenced by carotenoid polarity and interfacial location. Lutein and zeaxanthin contain hydroxyl groups and are more amphiphilic than β-carotene and lycopene. Their location near the interface can increase exposure to aqueous oxidants but could also facilitate transfer to bile-rich intestinal colloids. Esterification changes their polarity and can strongly influence micellization and analytical recovery (Dai et al., 2021; Jeong et al., 2021; Liu, Yan, et al., 2024; Ma et al., 2024; Mantovani et al., 2025; Nunes et al., 2023; Shu et al., 2023; Vu et al., 2022; Zhang, Zhang, et al., 2024).

Astaxanthin contains hydroxyl and keto groups and can be positioned across the interfacial region. This location allows it to participate directly in interfacial antioxidant reactions but also exposes it to reactive species from both the oil and aqueous phases. Free, esterified, and aggregated astaxanthin forms should therefore be considered separately (Saechio et al., 2023; Yang et al., 2017; Zhang, Xiang, et al., 2024; Zheng et al., 2024). These carotenoid-specific differences in emulsion localization, exposure to aqueous or interfacial oxidants, dependence on lipid hydrolysis, and transfer into mixed intestinal colloids are compared in Fig. 3.

**Fig. 3:**
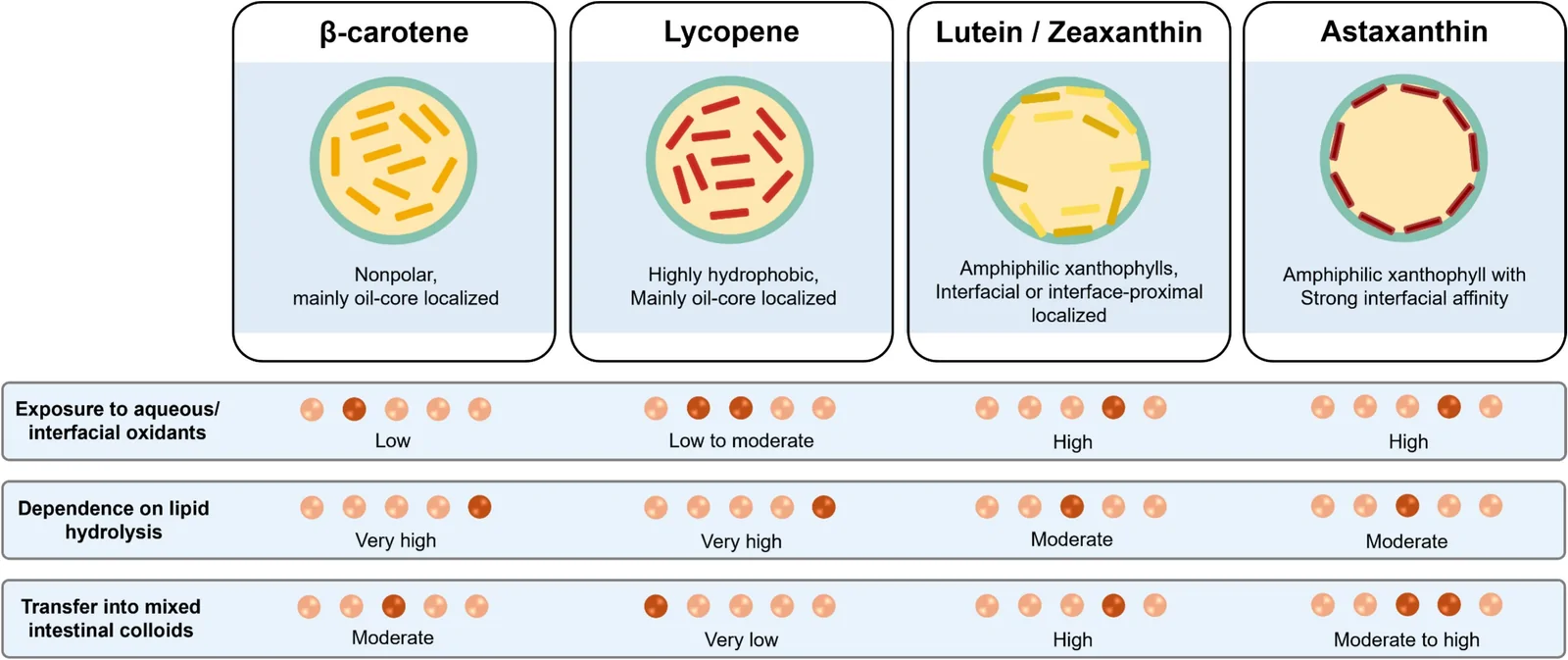
Carotenoid-specific localization and relative delivery behavior in plant protein-stabilized emulsions. β-Carotene and lycopene are highly nonpolar and are located predominantly within the oil core, whereas the amphiphilic xanthophylls lutein, zeaxanthin, and astaxanthin show greater interfacial or interface-proximal localization. These differences influence exposure to aqueous and interfacial oxidants, dependence on lipid hydrolysis, and transfer into mixed intestinal colloids. β-Carotene and lycopene have a high dependence on lipid digestion, but lycopene generally shows particularly limited transfer because of its high hydrophobicity. Lutein and zeaxanthin exhibit greater transfer into intestinal colloids, while astaxanthin shows intermediate-to-high transfer associated with its amphiphilic structure and strong interfacial affinity. The ratings represent qualitative relative tendencies rather than fixed quantitative values and may vary with carrier oil, carotenoid form, droplet structure, interfacial composition, and digestion conditions.

These carotenoid-specific differences are also influenced by carrier oil composition. Long-chain unsaturated oils can form intestinal colloids with high solubilizing capacity but are highly susceptible to oxidation. Medium-chain oils are more resistant to oxidation and are digested rapidly, yet their digestion products may solubilize highly hydrophobic carotenoids less effectively. Therefore, replacing polyunsaturated oil with a more stable oil can improve storage retention but lower micellization (Hart et al., 2018; Meroni & Raikos, 2018; Qian et al., 2012).

After micellization, cellular uptake provides additional information but should not be described as bioavailability. Caco-2 or mucus-producing co-culture models can determine uptake from the mixed-colloidal fraction, but the result depends on colloid composition, dose, incubation time, and cell condition. Oxidized lipid products can also affect cell viability and membrane integrity. Therefore, cellular experiments should follow complete chemical and colloidal characterization of the digesta (Cabezas-Terán et al., 2023; Hayes et al., 2022; Kotake-Nara & Nagao, 2012; Rošul et al., 2022; Xu et al., 2024; Yao et al., 2023).

## 5 Linking oxidative aging to digestion

The gastrointestinal behavior of carotenoid emulsion cannot be predicted only from the properties measured immediately after homogenization. Before consumption, the emulsion has normally been stored for a certain period. During this time, lipid hydroperoxides, aldehydes, oxidized proteins, carotenoid isomers, and degradation products can accumulate even when no visible phase separation is observed (Hennebelle et al., 2024; Meléndez-Martínez et al., 2023; Xu et al., 2013). Therefore, the oxidative condition immediately before digestion should be considered as an important factor affecting its subsequent gastrointestinal behavior.

Despite its importance, storage stability and digestive performance are often evaluated using separate experiments. A formulation may be stored for physical and chemical analysis, while a fresh batch is used for digestion. Such a design can show that the formulation is stable and digestible under two different conditions, but it cannot prove that the stored formulation remains digestible.

To address these limitations, this review proposes a storage-to-digestion strategy that considers preparation, storage, oxidation, gastrointestinal disassembly, lipid digestion, and carotenoid micellization as connected stages. The carotenoid recovered in mixed micelles depends on the losses occurring at all previous stages. Accordingly, studies using this proposed strategy should include time-resolved measurements, common sample batches, consistent calculations, and clear terminology. One important requirement is consistent calculation because carotenoid bioaccessibility can be calculated using different denominators. The denominator may be the initial carotenoid amount, the amount remaining before digestion, or the amount present in the intestinal digesta, and these choices can produce different values and interpretations (Geng, Feng, et al., 2023; Xavier & Mercadante, 2022).

### 5.1 Limitations of current approaches

#### 5.1.1 Storage and digestion are commonly evaluated separately

Formulations are often optimized using droplet size, creaming, encapsulation efficiency, or carotenoid retention during storage. Digestion is then evaluated using freshly prepared samples (Liu et al., 2021; Ma et al., 2022). This approach assumes that storage does not change pepsin susceptibility, bile salt displacement, or lipase adsorption. However, a protein layer that is initially digestible can become carbonylated and cross-linked during storage.

Even when storage and digestion are both reported in the same study, digestion is frequently conducted only at day zero. Therefore, a statement that the system has both high storage stability and high bioaccessibility may be based on two independent observations. Future studies should digest samples obtained from the same batches at the beginning, middle, and end of the intended shelf life.

This design would show whether digestive performance is maintained, temporarily improved, or gradually reduced during oxidative aging. It would also help distinguish moderate oxidation effects from severe deterioration.

#### 5.1.2 Oxidative aging is incompletely characterized

Physical stability is not a direct indicator of chemical stability. A similar droplet size and ζ-potential do not confirm that the interfacial protein layer and carotenoid remain chemically unchanged. Likewise, reversible flocculation can occur without severe oxidation. Therefore, the term stability should be specified as physical stability, lipid oxidative stability, protein oxidative stability, carotenoid retention, or digestive stability (Hennebelle et al., 2024; Xu et al., 2013).

For chemical stability assessment, lipid oxidation is often evaluated using a single measurement. This can be misleading because hydroperoxides may decline as they decompose, while TBARS provides only a nonspecific estimate of secondary oxidation. Therefore, at least one primary and one secondary oxidation measurement should be used together. Protein oxidation should also be included. Protein carbonyls and free sulfhydryl groups are minimum indicators, while dityrosine, molecular-weight distribution, and interfacial aggregation provide additional information (Hennebelle et al., 2024; Poojary & Lund, 2022).

Carotenoid analysis should also be included in chemical stability assessment and should resolve the parent compound and major isomers. Accelerated oxidation studies should also identify relevant degradation products when possible. Such detailed analysis is important because measurements of total carotenoids may not reveal changes in isomer composition or reductions in extraction recovery (Meléndez-Martínez et al., 2023).

Such accelerated storage conditions need careful interpretation. High temperature, radical initiators, or direct hydrogen peroxide addition can produce pathways that differ from normal food storage. Pre-oxidation of the protein, induced oxidation after emulsion preparation, and spontaneous storage oxidation answer different questions and should not be treated as equivalent (Chen et al., 2013; Li et al., 2021; Li, Cai, et al., 2022).

#### 5.1.3 Digestion and micelle separation methods differ among studies

The INFOGEST method has improved comparability by standardizing digestive fluid composition, enzyme activity, bile concentration, pH, temperature, and digestion time (Brodkorb et al., 2019). Nevertheless, carotenoid emulsion studies frequently modify the oral phase, gastric lipase, pancreatin activity, bile concentration, calcium, sample-to-fluid ratio, and digestion time. These changes can markedly affect lipolysis and micelle formation (Morelli & Rodriguez-Concepcion, 2022).

These modifications can be appropriate for a specific research question, but they should be fully reported and justified. Enzyme activity should be measured rather than estimated only from powder mass. Also, the actual lipid and protein load entering digestion should be stated because enzyme-to-substrate ratios influence the result (Tan et al., 2020).

Careful control of digestion conditions should be accompanied by appropriate evaluation of digestive responses. Proteolysis, lipolysis, and micellization should not be represented by a single endpoint. Incomplete protein digestion can explain poor lipase access, but free fatty acid release does not confirm carotenoid micellization. Likewise, a single endpoint measurement may not reveal differences in lag time and digestion rate. In this sense, kinetic data are especially useful for oxidized interfaces (Li et al., 2011; Mutsokoti et al., 2017).

Bioaccessibility assessment also depends strongly on the method used to collect the mixed-colloidal fraction. The optimal centrifugation conditions depend on the size and composition of the digesta (Liu et al., 2022). Insufficient centrifugation can leave oil droplets in the aqueous fraction, while excessive centrifugation or filtration can remove micelles and protein-associated colloids. Recovery before and after each separation step should be reported.

#### 5.1.4 Denominators and terms are not used consistently

Bioaccessibility can be calculated relative to the carotenoid originally added, the amount remaining before digestion, or the amount present in the total intestinal digesta. Using these different quantities as the denominator can produce different bioaccessibility values. None is necessarily incorrect, but the selected denominator must be stated clearly (Geng, Feng, et al., 2023; Xavier & Mercadante, 2022).

Each denominator reflects a different stage of the delivery process. Using the initial formulation concentration describes the total delivery from production through storage and digestion. Using the concentration immediately before digestion describes the digestive performance of the aged sample. Using the total intestinal digesta concentration describes the fraction transferred into the aqueous colloidal phase after digestion.

Clear interpretation also requires consistent use of terminology. The terms retention, release, micellization, uptake, bioaccessibility, and bioavailability should be distinguished from one another. Cellular uptake is a later step than bioaccessibility and does not demonstrate systemic bioavailability. Bioavailability requires evidence of absorption and distribution in an animal or human model (Geng, Bao, et al., 2023; Xavier & Mercadante, 2022).

### 5.2 A storage-to-digestion evaluation strategy

#### 5.2.1 Initial ingredient and emulsion characterization

The carrier oil should be analyzed for fatty acid composition, tocopherols, and initial hydroperoxides. Plant protein ingredients should be characterized for protein content, solubility, particle size, protein carbonyls, free sulfhydryls, residual lipids, phenolic compounds, phytate, and soluble iron and copper. These values determine the initial chemical condition of the formulation (Hennebelle et al., 2024; Münch, Schroën, & Berton-Carabin, 2024).

After ingredient characterization, the fresh emulsion should be evaluated for droplet-size distribution, ζ-potential, interfacial protein concentration, carotenoid recovery, carotenoid isomer composition, lipid oxidation, protein oxidation, and microstructure (Drusch et al., 2021; Hennebelle et al., 2024; Meléndez-Martínez et al., 2023). Interfacial rheology can be added when available. Relative storage retention should not be compared among formulations that have different initial carotenoid recovery or initial oxidation.

#### 5.2.2 Controlled and time-resolved storage

Storage temperature, light, oxygen exposure, container geometry, headspace, closure type, and opening frequency should be controlled (Hennebelle et al., 2024). At least three time points are recommended: the beginning, an intermediate point, and the end of storage. More time points are required when oxidation kinetics are a major objective. Microbial growth should also be controlled or monitored during long-term storage because microbial deterioration can alter the emulsion. Microbial proteases and lipases can hydrolyze proteins and lipids, respectively, and these changes may interfere with the interpretation of oxidation-related changes. Therefore, microbiological changes should be monitored to distinguish microbial deterioration from oxidative aging during storage (Snyder et al., 2024).

At each storage time point, the same analytical panel should be used. A minimum panel includes droplet size, primary lipid oxidation, secondary lipid oxidation, protein carbonyls, free sulfhydryl groups, intact carotenoid, and major carotenoid isomers. Advanced analysis can include volatile compounds, aldehyde-protein adducts, LC-MS carotenoid products, and interfacial rheology (Hennebelle et al., 2024; Meléndez-Martínez et al., 2023; Poojary & Lund, 2022).

To ensure that changes across these time points reflect oxidative aging rather than batch variation, samples for storage analysis and digestion should come from the same containers or identically treated replicate containers. This prevents batch variation from being interpreted as an effect of oxidative aging.

#### 5.2.3 Digestion of stored emulsions

Samples collected at each storage time should be digested using the same protocol. The INFOGEST method is suitable for standardized comparisons unless a specific modification is justified (Brodkorb et al., 2019). The method standardizes digestive fluid composition, enzyme activity, bile concentration, pH, temperature, and digestion time across the oral, gastric, and intestinal phases. However, as a static model, it does not reproduce dynamic physiological processes such as gastric emptying, continuous secretion of digestive fluids, or intestinal absorption. Thus, the method is useful for controlled comparison among stored emulsions but does not fully reproduce *in vivo* gastrointestinal digestion.

When using the INFOGEST method to evaluate the digestion of stored emulsions, digesta should be collected at the end of gastric digestion and at several time points during intestinal digestion. Gastric measurements should include protein hydrolysis, particle size, microstructure, and carotenoid retention. Intestinal analysis should include lipolysis kinetics, colloidal structure, carotenoid retention, and micellization. A fresh emulsion and carotenoid dissolved in the same oil are useful controls. In addition, a stored emulsion without carotenoids can be used to evaluate the effect of carotenoids on oxidation because carotenoids are lipophilic compounds with radical-scavenging activity and can influence oxidation kinetics at the oil–water interface (El-Agamey et al., 2004; Young & Lowe, 2001). Comparison with the corresponding carotenoid-containing emulsion can show how the presence of carotenoids affects oxidative changes during storage. Furthermore, an oxidized oil control can be prepared using pre-oxidized oil with non-oxidized protein to evaluate the effect of lipid oxidation. In contrast, a pre-oxidized protein control can be prepared using oxidized protein with fresh oil to evaluate the effect of protein oxidation. A low-oxidation oil control can also help distinguish the contribution of carrier oil quality from that of the plant protein interface. Together, these controls can help distinguish the contributions of carotenoids, carrier oil quality, lipid oxidation, and protein oxidation to changes in digestion and carotenoid behavior.

#### 5.2.4 Fraction-specific mass balance

After intestinal digestion, the carotenoid should be measured in the total digesta and in the separated fractions. These fractions can include surface oil, undigested oil, sedimented material, aqueous mixed colloids, filter-retained material, and material remaining on the digestion vessel. A mass balance can show whether the carotenoid was degraded, sedimented, retained in oil, bound to protein aggregates, or transferred into the aqueous phase.

To quantify these changes across storage and digestion, four values can be used: storage retention, digestive retention, post-storage micellization efficiency, and overall delivery efficiency. Together, these values can identify whether the major limitation occurs during storage, during digestion, or at both stages. The storage retention (%) can be calculated using Eq. (1):

(1) $$\mathrm{Storage retention}\left(\%\right)=\frac{\mathrm{Intact carotenoid immediately before digestion}}{\mathrm{Intact carotenoid}\;\mathrm{at}\;\mathrm{the beginning of storage}}\times 100$$

The digestive retention (%) can be calculated using Eq. (2):

(2) $$\mathrm{Digestive retention}\left(\%\right)=\frac{\mathrm{Intact carotenoid in total intestinal digesta}}{\mathrm{Intact carotenoid immediately before digestion}}\times 100$$

The post-storage micellization efficiency (%) can be calculated using Eq. (3):

(3) $$\mathrm{Post}-\mathrm{storage micellization efficiency}\left(\%\right)=\frac{\mathrm{Intact carotenoid in the mixed colloidal fraction}}{\mathrm{Intact carotenoid in total intestinal digesta}}\times 100$$

The overall delivery efficiency (%) can be calculated using Eq. (4):

(4) $$\mathrm{Overall delivery efficiency}\left(\%\right)=\frac{\mathrm{Intact carotenoid in the mixed colloidal fraction}}{\mathrm{Intact carotenoid}\;\mathrm{at}\;\mathrm{the beginning of storage}}\times 100$$

For example, if an emulsion contains 100 μg of intact carotenoid at the beginning of storage, 80 μg immediately before digestion, 60 μg in the total intestinal digesta, and 30 μg in the mixed-colloidal fraction, the storage retention, digestive retention, post-storage micellization efficiency, and overall delivery efficiency are 80%, 75%, 50%, and 30%, respectively. These values indicate that 20% of the initial carotenoid is lost during storage and that additional loss occurs during digestion. The overall delivery efficiency further shows that 30% of the carotenoid present at the beginning of storage is ultimately recovered in the mixed-colloidal fraction. Thus, these equations can be used together to determine at which stage carotenoid loss occurs during storage and gastrointestinal digestion.

After these stage-specific measurements, if cellular uptake is evaluated, the mixed-colloidal fraction should first be characterized as carotenoid concentration, particle size, bile salts, protein aggregates, and oxidized lipid products. Cell viability and membrane integrity controls are required because oxidized digesta can affect the cells independently of carotenoid uptake.

For practical implementation, considering analytical cost and accessibility, a two-tier strategy can be used. The first tier includes minimum measurements needed to track major changes during storage and digestion, whereas the second tier includes advanced analyses for more detailed investigation of oxidation products, interfacial changes, and carotenoid transformation. A stage-specific storage-to-digestion evaluation strategy, including minimum and advanced measurements, controls, reporting requirements, and primary outputs, is summarized in Table 3.

**Table 3:** Recommended storage-to-digestion evaluation strategy for plant protein-stabilized carotenoid emulsions.

Evaluation stage	Minimum measurements	Advanced/confirmatory measurements	Controls and reporting requirements	Primary interpretation or output
Ingredient baseline	Oil fatty acid composition and initial hydroperoxides; protein content, solubility, particle size, carbonyls, free sulfhydryls, residual lipids, phenolics, phytate, and soluble Fe/Cu.	Oil tocopherols; phospholipids; pre-existing volatile/secondary oxidation products; protein molecular-weight distribution and surface hydrophobicity.	Report botanical source, fractionation and drying history, ingredient storage, water and salt sources, and analytical basis for protein concentration.	Defines the initial prooxidant/antioxidant inventory and prevents botanical source or protein purity from being used as the sole explanatory variable.
Fresh emulsion	Droplet-size distribution, ζ-potential, carotenoid recovery/isomers, primary and secondary lipid oxidation, protein carbonyls/free sulfhydryls, and microstructure.	Adsorbed protein, interfacial tension and dilatational rheology, spatial/fraction-specific oxidation, carotenoid oxidation products.	Include carotenoid-in-oil and fresh emulsion controls; state oil fraction, protein loading, homogenization conditions, pH, ionic strength, and oxygen exposure.	Establishes the true day-zero condition and interfacial coverage; initial recovery differences must be considered before comparing retention.
Time-resolved storage	At least beginning, intermediate, and end points; repeat the same panel for droplet size, lipid oxidation, protein oxidation, intact carotenoid, and major isomers.	Volatile aldehydes, aldehyde-protein adducts, LC-MS oxidation products, interfacial rheology, oxygen consumption, and spatial imaging.	Control temperature, light, oxygen/headspace, container geometry, closure, and opening frequency. Use the same containers or identically treated replicates for analysis and digestion.	Distinguishes mild, intermediate, and severe oxidative aging and links chemical remodeling to later digestive behavior.
Gastric digestion of stored samples	Protein hydrolysis, particle-size distribution, microstructure, phase separation, and carotenoid retention after the gastric stage.	Pepsin diffusion, soluble peptide release, adsorbed peptide analysis, gastric lipolysis, and fractionation into soluble/dispersed/sedimented material.	Use the same digestion protocol for every storage point; report enzyme activities, pH, times, sample-to-fluid ratio, and actual protein/lipid load.	Determines whether oxidation exposes cleavage sites or instead creates cross-linked barriers and physical sequestration.
Intestinal digestion	Lipolysis kinetics (lag, initial rate, plateau), particle/microstructure changes, carotenoid retention, and mixed-colloidal carotenoid.	Direct FFA/MAG/TAG analysis; bile-salt displacement; SEC, AF4, cryo-TEM, or scattering to identify colloid types.	Report bile, calcium, pancreatin/lipase activities, pH-stat assumptions, and timing. Include fresh, low-oxidation oil, oxidized oil, and pre-oxidized protein controls when mechanism separation is needed.	Shows whether reduced delivery results from poor lipase access, insufficient digestion products, colloidal precipitation, or carotenoid degradation.
Fraction-specific mass balance	Measure intact carotenoid in total digesta and aqueous mixed-colloidal fraction; record sediment and visible/undigested oil.	Analyze filter-retained material, vessel residues, protein-bound carotenoid, surface oil, and oxidation products.	Report centrifugation force/time/temperature, filter material/pore size, and recovery after every separation step.	Calculate storage retention, digestive retention, post-storage micellization efficiency, and overall delivery efficiency using explicitly stated denominators.
Optional cellular uptake	Carotenoid concentration of the applied mixed-colloidal fraction, uptake per cell/protein, cell viability, and membrane-integrity controls.	Characterize colloid size, bile salts, protein aggregates, and oxidized lipid products; use mucus-producing co-culture where relevant.	Do not label cellular uptake as bioavailability. Match dose and exposure across storage groups and verify that oxidized digesta is not independently cytotoxic.	Provides a post-bioaccessibility uptake estimate after chemical and colloidal confounders have been resolved.

AF4 indicates asymmetric-flow field-flow fractionation; cryo-TEM indicates cryogenic transmission electron microscopy; FFA indicates free fatty acids; MAG indicates monoacylglycerols; SEC indicates size-exclusion chromatography; TAG indicates triacylglycerols. Representative references supporting the recommended measurements and digestion procedures include Brodkorb et al. (2019), Mutsokoti et al. (2017), Hennebelle et al. (2024), and Brüls-Gill et al. (2024).

### 5.3 Balancing stability and digestive release

#### 5.3.1 Selection of plant protein and carrier oil

The goal is not to form the strongest possible interfacial protein layer. An excessively persistent layer can protect carotenoids during storage but remain difficult to digest. By contrast, a readily digestible interface can provide high bioaccessibility when fresh but poor protection during shelf life. The desirable system should be stable under storage conditions and responsive under gastrointestinal conditions (Yan et al., 2024).

To achieve this balance, protein selection should include oxidative composition as well as emulsifying activity. A high-purity isolate may contain soluble metals or pre-oxidized protein, whereas a concentrate may retain natural chelators and phenolic compounds (Münch, Schroën, & Berton-Carabin, 2024; Münch, Stoyanov, et al., 2024). Protein blends can combine rapid adsorption with a stronger interfacial protein layer, but their cross-linking and digestibility should be evaluated.

After protein selection, controlled modification using heat, ultrasound, or pH shifting can improve solubility and adsorption. However, excessive treatment can generate aggregates and expose oxidation-sensitive residues. Therefore, modified proteins should be optimized based on storage-to-digestion performance, with droplet size used as one of several evaluation criteria (Jiang et al., 2022; Ling et al., 2023; Zhang et al., 2021; Zhang, Wei, et al., 2024).

Carrier oil selection requires a similar balance between storage stability and digestive performance. Long-chain unsaturated oils can support carotenoid micellization but are oxidation-sensitive. Medium-chain oils are more stable and rapidly digested, but they may provide lower solubilizing capacity (Hennebelle et al., 2024; Qian et al., 2012). Blending oils can be useful, but the optimal composition depends on carotenoid structure and the desired shelf life. The physical state of the lipid should also be considered. For example, nanostructured lipid carriers contain both solid and liquid lipids, producing a partially crystalline lipid phase that differs from the fully liquid oil droplets of conventional emulsions. This difference in physical state can alter lipid mobility, oxidation behavior, and lipase accessibility during digestion. Thus, lipid composition and physical state can both influence carotenoid stability and digestive release (Hart et al., 2018; Shu et al., 2023).

#### 5.3.2 Interfacial antioxidants and digestively responsive structures

Antioxidant effectiveness depends on location. Hydrophilic antioxidants remain mainly in water, strongly lipophilic compounds stay in the oil, and amphiphilic compounds can concentrate at the interface. Because many oxidation reactions occur near the droplet surface, interfacial localization may be more important than total concentration (Geng, Feng, et al., 2023; Gong et al., 2024; Guo et al., 2022; Huang et al., 2024).

Metal chelators provide another approach to controlling oxidation by reducing the catalytically active metal fraction without producing soluble redox-active complexes. Their interaction with proteins should also be considered because chelation can change protein charge and conformation. Phenolic compounds may scavenge radicals but can form strong non-covalent or covalent associations with proteins and reduce proteolysis (Li, Huang, et al., 2023).

Reversible protein-polysaccharide interactions can also help balance storage protection and digestibility without permanent cross-linking. A complex can increase interfacial thickness during storage and then dissociate when pH and ionic strength change during digestion. In soy protein-citrus pectin systems, stronger protective structures improved storage behavior, but the formulation with the highest apparent protection did not always provide the highest β-carotene release (Huang, Yan, et al., 2022; Li, Chen, et al., 2023; Ma et al., 2022; Nooshkam & Varidi, 2021; Yang et al., 2026; Zhang et al., 2023).

These observations suggest that the ideal interface should limit oxygen and metal access during storage but allow pepsin hydrolysis, bile salt displacement, or intestinal reorganization. This conditionally stable interface is more appropriate than an interface designed only for maximum mechanical strength. This desired transition from storage protection to digestive disassembly is illustrated in Fig. 4.

**Fig. 4:**
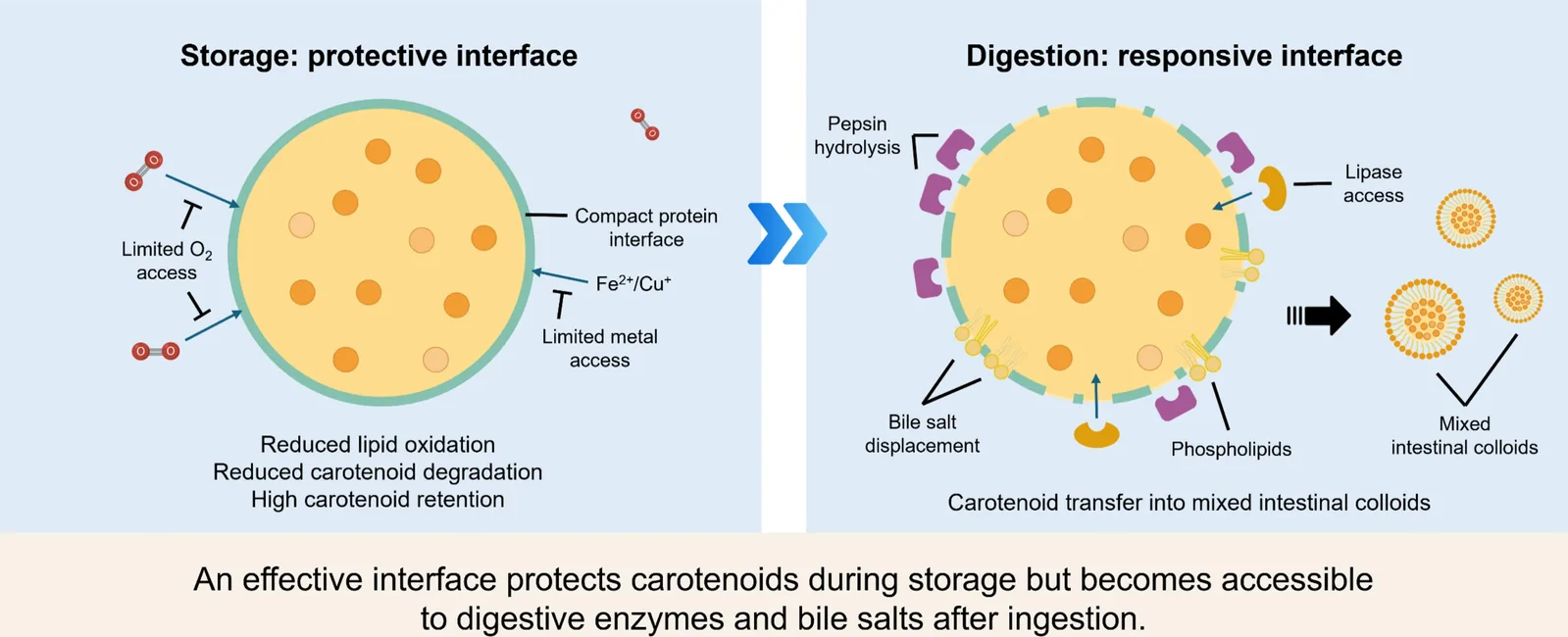
Desired interfacial behavior of plant protein-stabilized carotenoid emulsions during storage and gastrointestinal digestion. During storage, a compact plant protein interface limits the access of oxygen and prooxidant metal ions to the oil phase, thereby reducing lipid oxidation and carotenoid degradation. During gastrointestinal digestion, pepsin hydrolysis and bile salt displacement disrupt the protein interface and increase lipase access to the lipid phase. Lipid digestion and interfacial disassembly then facilitate carotenoid transfer into mixed intestinal colloids containing bile salts, phospholipids, and lipid digestion products.

#### 5.3.3 Multi-objective evaluation

Most formulation studies optimize one response, such as minimum droplet size, maximum carotenoid retention, or maximum fresh sample bioaccessibility. These responses can select different formulations. Therefore, multi-objective evaluation is required.

This multi-objective evaluation can result in four general outcomes. A system can show low oxidative stability and low post-storage micellization efficiency, high oxidative stability and low post-storage micellization efficiency, low oxidative stability and high post-storage micellization efficiency, or high oxidative stability and high post-storage micellization efficiency. The fourth outcome is the desired target because the carotenoid is preserved and then released effectively.

To achieve this target, future studies should report the absolute amount of intact carotenoid delivered after realistic storage and evaluate bioaccessibility using stored samples. Shelf-life-adjusted delivery efficiency provides a more practical basis for food design because it includes both chemical preservation and digestive transfer.

## 6 Conclusions and future perspectives

Plant protein-stabilized emulsions are useful for incorporating carotenoids into aqueous and plant-based foods, but their performance cannot be assessed using only freshly prepared samples. During storage, lipids, interfacial proteins, carotenoids, metals, phenolics, and other minor components undergo interconnected reactions that alter the oil-water interface.

In this review, oxidative aging describes these storage-dependent changes, including lipid oxidation, protein carbonylation and cross-linking, as well as carotenoid isomerization and cleavage. Consequently, the aged interface may differ from the fresh interface in charge, flexibility, hydration, and digestive susceptibility.

The effects of oxidation are degree dependent. Studies of soy and rice bran proteins show that moderate protein oxidation can improve interfacial properties, whereas extensive oxidation promotes rigid aggregation and reduces digestive accessibility (Chen et al., 2013; Li et al., 2021; Zhou et al., 2023). Thus, storage protection and digestive disassembly must be optimized together.

Future studies need to analyze and digest the same samples throughout storage and evaluate lipid and protein oxidation, carotenoid transformation, proteolysis, lipolysis, and micellization as connected stages. Standardized characterization of protein source, fractionation history, residual components, transition metals, and initial oxidation is also required.

In addition to these methodological improvements, studies should extend from emulsions prepared under controlled laboratory conditions with plant proteins, carrier oils, and carotenoids to actual food products, where other ingredients and processing conditions can alter both oxidation and digestion. Dynamic gastrointestinal models, intestinal cell models, animal studies, and human studies are needed to determine whether changes observed in static *in vitro* digestion are reflected in carotenoid absorption. In addition, the effects of industrial processing, packaging, and realistic shelf-life conditions should be considered to translate these findings into the development of plant-based foods with improved carotenoid delivery.

Ultimately, the target interface protects carotenoids during shelf life but undergoes degradation or displacement during digestion. Shelf-life-adjusted delivery efficiency is proposed as a measure that integrates storage retention with digestive transfer after storage.

## CRediT authorship contribution statement

**Uyory Choe:** Writing – review & editing, Writing – original draft, Visualization, Methodology, Investigation, Funding acquisition, Conceptualization.

## Funding

This work was supported by the 10.13039/501100003725National Research Foundation of Korea (NRF) grant funded by the Korean government (MSIT) (Grant No. RS-2026-25485983). This work was also supported by 10.13039/501100002641Konkuk University (Grant No. 2025-A019–0069).

## Declaration of competing interest

The author declares that there are no known competing financial interests or personal relationships that could have appeared to influence the work reported in this paper.

## Data Availability

No data was used for the research described in the article.
